# Association between BDNF Gene Polymorphisms and Serotonergic Activity Using Loudness Dependence of Auditory Evoked Potentials in Healthy Subjects

**DOI:** 10.1371/journal.pone.0060340

**Published:** 2013-04-09

**Authors:** Young-Min Park, Seung-Hwan Lee, Heon-Jeong Lee, Seung-Gul Kang, Jung-Ah Min, Jeong-Ho Chae

**Affiliations:** 1 Department of Psychiatry, Inje University, Ilsan Paik Hospital, Goyang, Republic of Korea; 2 Clinical Emotion and Cognition Research Laboratory, Goyang, Republic of Korea; 3 Department of Psychiatry, Korea University College of Medicine, Seoul, Republic of Korea; 4 School of Medicine, Gachon University, Incheon, Republic of Korea; 5 Department of Psychiatry, Seoul St. Mary’s Hospital, College of Medicine, The Catholic University of Korea, Seoul, Republic of Korea; Charité-Universitätsmedizin Berlin, Germany

## Abstract

It has been proposed that the loudness dependence of auditory evoked potentials (LDAEP) would be a reliable indicator of central serotonin system activity in humans. Serotonin levels and turnover are also increased by brain-derived neurotrophic factor (BDNF). The aim of the present study was to determine whether there is an association between genetic polymorphisms of *BDNF* and the LDAEP in healthy Korean young adults. The cohort comprised 211 mentally and physically healthy subjects, all of whom were nonsmokers (111 males, 100 females; age: 20∼32 years). To avoid hormonal effects, the LDAEP was measured during days 2–5 after the beginning of menstruation for female subjects. In addition, *BDNF* polymorphisms (rs6265, rs2030324, and rs1491850) were genotyped. The strength of the LDAEP differed significantly among the *BDNF* genotype groups. Furthermore, the distribution of genotypic frequencies differed significantly between subjects with high and low LDAEPs. In particular, subjects with the Val/Met (A/G) genotype for rs6265, the T/T genotype for rs2030324, or the C/C genotype for rs1491850 had a higher LDAEP, indicating lower central serotonergic activity. A low LDAEP was more prevalent than a high LDAEP among those with the C-T haplotype (C genotype for rs2030424 and T genotype for rs1491850). Our results concur with previous findings on *BDNF* polymorphisms and serotonergic drug responses in psychiatric disorder patients. The present results suggest the possibility that *BDNF* polymorphisms and LDAEP patterns can predict altered serotonergic activity.

## Introduction

It has been proposed the loudness dependence of auditory evoked potentials (LDAEP) is a reliable indicator of central serotonin system activity in humans [1], [2]. The LDAEP reflects the change in the auditory evoked N1/P2 component evoked by an increase in stimulus intensity and has been found to be inversely associated with central nervous system serotonergic activity [3], such that a weak LDAEP reflects high serotonergic neurotransmission, and vice versa [4].

From these findings it has been proposed that the LDAEP is a biological marker of central serotonergic activity in major depressive disorder and other psychiatric diseases [1], [5], [6]. In other words, a significant correlation has been found between a strong LDAEP – indicating low serotonergic function – and a favorable response to selective serotonin reuptake inhibitors (SSRIs) in patients with major depressive disorder or generalized anxiety disorder [7], [8], [9], [10], [11], [12].

Brain-derived neurotrophic factor (BDNF) is known to play a role in neuronal survival and plasticity and to be required for proper development and survival of dopaminergic, GABAergic, cholinergic, and serotonergic neurons [13]. It was found that BDNF increases both the serotonin levels and turnover of serotonin [14]. Chronic antidepressant drug therapy up-regulates the expression of BDNF and its receptor and increases neurogenesis in the adult rat hippocampus [15], [16], [17]. There is also evidence that acute treatment rapidly activates TrkB (tropomyosin-related kinase B) receptors, which are related to BDNF [18]. This effect is not observed in serotonin-depleted mice, which points to the crucial role of serotonin in increasing the actions of BDNF on its receptor [18].

Low serum BDNF levels are reportedly associated with a strong LDAEP as a reflection of low central serotonergic activity [19]. Recently, another study found an association between *BDNF* single-nucleotide polymorphisms (SNPs; rs6265-rs2030324-rs1491850) and the LDAEP as an indicator of central nervous serotonergic activity, as revealed in both haplotype and single-marker analyses in German descendents [20]. The haplotype analysis revealed that the LDAEP was stronger in carriers of the G(Val)-C-T [rs6265(Val66Met)-rs2030324-rs1491850] haplotype within the *BDNF* gene than in other haplotype carriers [20]. From this it was suggested that subjects with the *BDNF* haplotype G(Val)-C-T are characterized by low serotonergic activity and possibly by low serum BDNF levels [20]. The aim of this study was thus to determine the association between genetic polymorphisms of BDNF and the LDAEP in a healthy Korean population.

## Materials

### Subjects

Unrelated healthy young adults (age: 20–32 years) were recruited by advertisements from the general population of Goyang and Seoul, Korea. They were native Korean, and their parents were both Korean. Subjects were invited to a comprehensive interview, which included applying the Structured Clinical Interview for the Diagnostic and Statistical Manual of Mental Disorders, Fourth Edition (SCID I and SCID II) in order to exclude current and/or lifetime Axis I and II disorders [21], [22]. Subjects with a hearing problem, organic brain disease, or family history of a mental disorder were also excluded. All subjects were no smoking, and right handed. Finally, 211 healthy subjects (111 males, 100 females; age, 24.09±3.23 years, mean±SD; age range, 20–32 years) were included and submitted to electrophysiological recording and blood sampling for genotyping. Written informed consent to participate was obtained from all subjects, and the study protocol was approved by the both Ethics Committees of Inje University Ilsan Paik Hospital, and Seoul Saint Mary’s hospital, Catholic University.

### Electrophysiological Assessment and Amplitude Analysis

To avoid the hormonal effects on LDAEP, LDAEP measurement was conducted during 2^nd^ to 5^th^ day after the beginning of menstruation for female subjects [23]. Each subject was seated in a comfortable chair in a sound-attenuated room. The auditory stimulation comprised 1000 stimuli with an interstimulus interval randomized to between 500 and 900 ms. Tones of 1000 Hz and 80-ms duration (with a 10-ms rise and 10-ms fall) were presented at five intensities (55, 65, 75, 85, and 95 dB SPL) via headphones (MDR-D777, Sony, Tokyo, Japan). These stimuli were generated by E-Prime software (Psychology Software Tools, Pittsburgh, PA, USA). EEG data were recorded from 32 scalp sites using silver/silver-chloride electrodes according to the international 10–20 system (impedance <10 kΩ) and using an Auditory Neuroscan NuAmp amplifier (Compumedics USA, El Paso, TX, USA). Data were collected at a sampling rate of 1000 Hz using a bandpass filter form 0.5 to 100 Hz. In addition, four electrodes were used to measure both horizontal and vertical electrooculograms.

Data were reanalyzed using Scan 4.3 software with a bandpass filter from 1 to 30 Hz, and ocular contamination was removed using standard blink correction algorithms [24]. Event-related potential sweeps with artifacts exceeding 70 µV were rejected at all electrode sites. For each intensity and for each subject, the N1 peak (most-negative amplitude between 80 and 130 ms after the stimulus) and P2 peak (most-positive peak between 130 and 230 ms after the stimulus) were then determined at the Fz, Cz, Pz, C3, and C4 electrodes.

The peak-to-peak N1/P2 amplitudes were calculated for the five stimulus intensities, and the LDAEP was calculated as the slope of the linear regression.

### Genotyping

We chose to investigate the frequencies of three SNPs (rs6265, rs2030324, and rs1491850) of *BDNF*. Blood samples (5–10 ml) were collected into EDTA tubes, and genomic DNA was isolated using a NucleoSpin Blood DNA Extraction Kit (Macherey-Nagel, Düren, Germany) according to the manufacturer’s instructions. Genotyping was performed using high-resolution melting-curve analysis. Polymerase chain reactions (PCRs) were performed using a volume of 20 µl per reaction in a 96-well Bio-Rad CFX96 real-time PCR System (Bio-Rad, Hercules, CA, USA). Reaction mixtures included 1.5 µl of genomic DNA as a template, each of the *BDNF* primers at 200 mM (rs6265, forward: 5′-GAC ATC ATT GGC TGA CAC TT-3′, reverse: 5′-CGA ACT TTC TGG TCC TCA TC-3′; rs2030324, forward: 5′-CAA ACA TCA CAC AGC CTA AAT AG-3′, reverse: 5′-AGG ACA TTG AAT CAG ATG AAA GA-3′; rs1491850, forward: 5′-ATA AAG CTC ATA GAG TTG ATA ATC ATA CA-3′, reverse: 5′-CCC TCA AAG GCT GTC CAA-3′; BMS, Daejeon, South Korea), 1×Sso Fast EvaGreen SuperMix (Bio-Rad), and sterile H_2_O. The amplification protocol started at 98°C for 3 min, followed by 39 cycles of 98°C for 10 s and 58°C for 20 s. After an initial step of 95°C for 10 s and 65°C for 10 s, melting curves were generated between 65°C and 95°C, with increments of 0.3°C per cycle. High-resolution melting-curve profiles were analyzed with Bio-Rad Precision Melt Software.

### Statistical Analysis

All of the analyses were performed using standard software (SPSS for Windows). The presence of Hardy-Weinberg equilibrium was tested with the χ^2^ test for goodness of fit. Categorical data were also analyzed by using the χ^2^ test, and differences for continuous variables were evaluated using Student’s *t*-test or ANOVA or MANOVA. The level of statistical significance was set at *p*<0.05. We performed haplotype-based case-control analysis of the three SNPs. Haplotype and linkage disequilibrium analyses were performed using the software SNPAlyze version 7 (DYNACOM, Yokohama, Japan). The overall distribution of haplotypes was analyzed using 2×*m* contingency tables, with the level of statistical significance set at *p*<0.05. The *p* value of each haplotype was determined using χ^2^ analysis, the permutation method, and SNPAlyze version 7.

## Results

**Figure 1:**
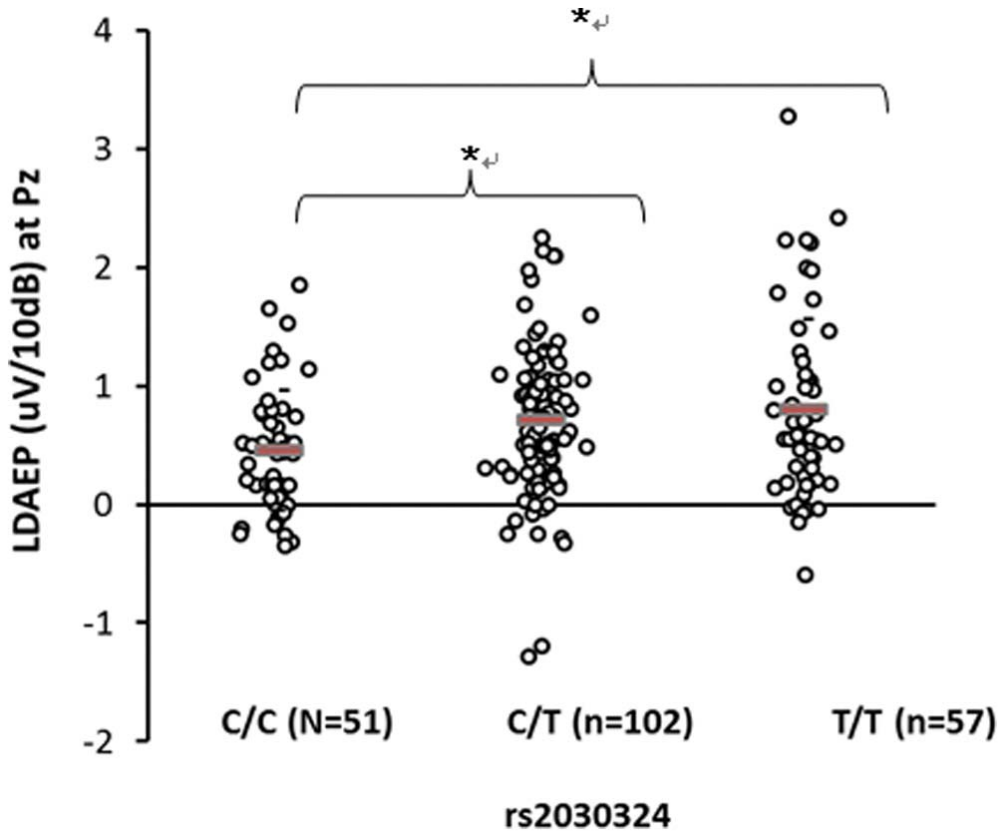
Comparison of LDAEP at Pz among genotypes of rs2030324. Mean values were presented as horizontal bars. There was a significant difference among 3 genotype groups (ANOVA) (p<0.05). There was a statistically significant difference between 2 genotype groups (C/C vs C/T, C/C vs T/T) (post-hoc analysis; LSD) (p<0.05).

**Figure 2:**
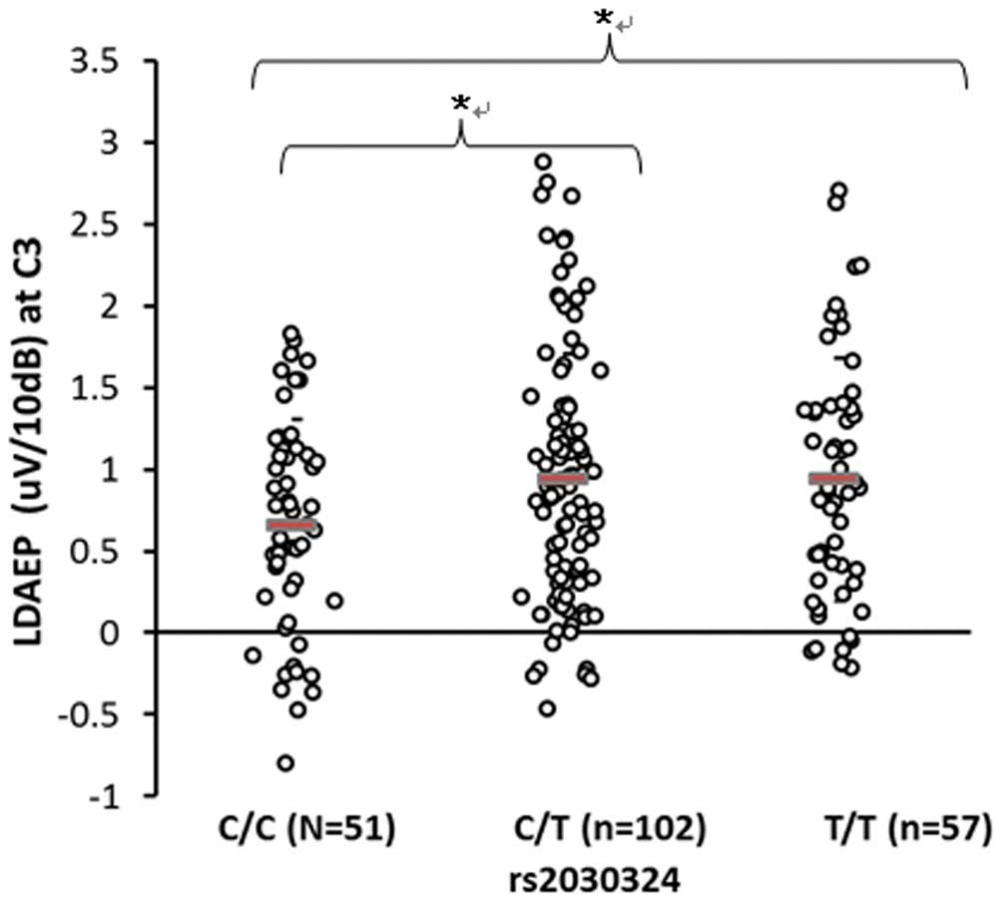
Comparison of LDAEP at C3 among genotypes of rs2030324. Mean values were presented as horizontal bars. There was a significant difference among 3 genotype groups (ANOVA) (p<0.05). There was a statistically significant difference between 2 genotype groups (C/C vs C/T, C/C vs T/T) (post-hoc analysis; LSD) (p<0.05).

**Figure 3:**
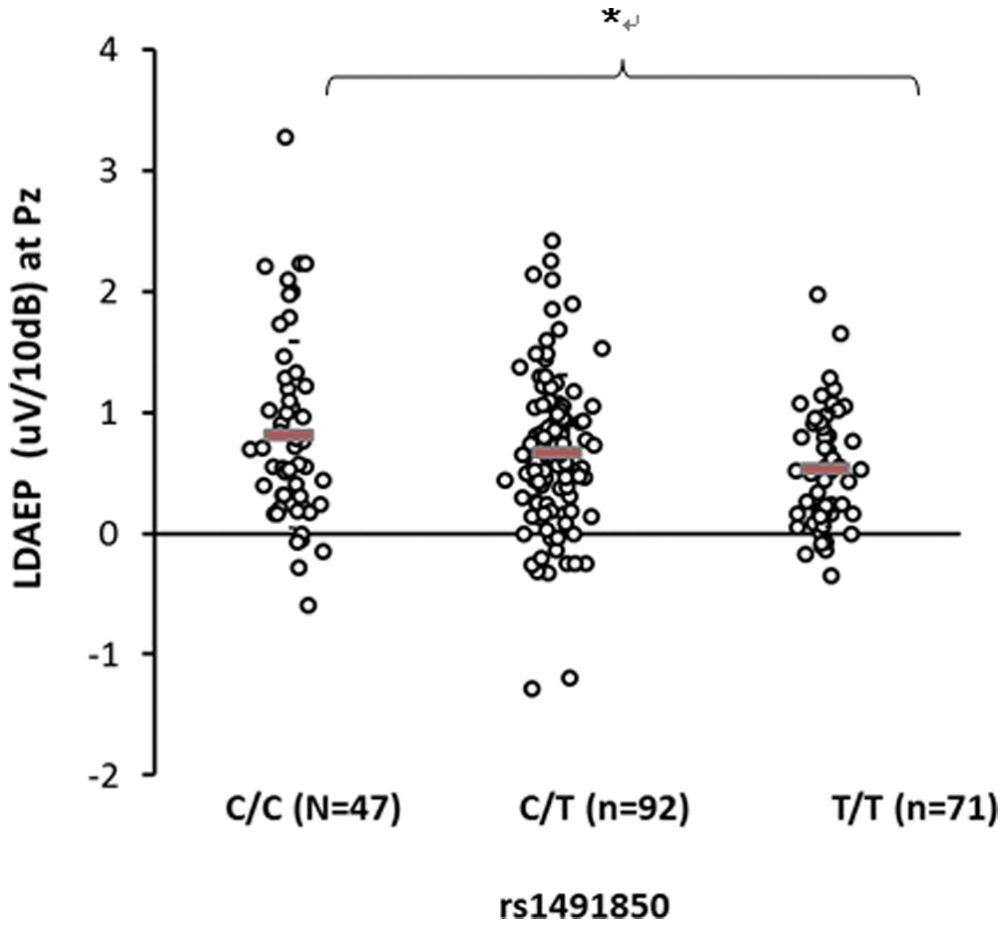
Comparison of LDAEP at Pz among genotypes of rs1491850. Mean values were presented as horizontal bars. There was a significant difference among 3 genotype groups (ANOVA) (p<0.05). There was a statistically significant difference between 2 genotype groups (C/C vs T/T) (post-hoc analysis; LSD) (p<0.05).

The genotypic frequencies of the three *BDNF* gene polymorphisms conformed with Hardy-Weinberg equilibrium. There were statistical analyses on demographics as well as allele frequencies of the three SNPs in *BDNF* gene (Table S1). ANOVA revealed that the LDAEP differed significantly among the genotype groups of rs2030324 at Pz (*F* = 4.829, df = 2, *p* = 0.009) and C3 (*F* = 3.285, df = 2, *p* = 0.039; Figure 1, 2 and Table 1). In addition, there was a marginally significant difference in the LDAEP among these genotype groups at Cz (*p* = 0.064; Table 1). The LDAEP differed significantly among the genotype groups of rs1491850 at Pz (*F* = 3.149, df = 2, *p* = 0.045; Figure 3 and Table 1). However, the LDAEP did not differ significantly among the genotype groups at any of electrode sites with regard to the genotypes of rs6265 (Table 1). MANOVA revealed no significant differences among the three genotype groups at five electrodes (Table S2). However, there were statistically significant differences between pairs of genotype groups in rs1491850 (C/C vs T/T at Pz) and rs2030324 (C/C vs T/T and C/T vs T/T at Cz, C/C vs T/T and C/T vs T/T at Pz, and C/C vs T/T and C/T vs T/T at C3) (post-hoc analysis; LSD) (*p*<0.05) (Figures 1, 2, and 3).

**Table 1:** Statistical analyses on the intensity of LDAEP with genotypes of the three SNPs in BDNF gene and the distribution of genotypic frequencies among healthy subjects with low and high (L/H) LDAEP of five electrodes.

BDNF marker	Genotype	LDAEP at Cz (L/H)	LDAEP at Pz (L/H)	LDAEP at Fz (L/H)	LDAEP at C3 (L/H)	LDAEP at C4 (L/H)
rs6265	Val/Val (48)	1.12±0.86 (22/26)	0.66±0.75 (27/21)	0.60±0.67 (28/20)	0.86±0.70 (23/25)	0.91±0.71 (36/31)
	Val/Met (95)	1.17±0.88 (44/51)	0.75±0.64 (38/57)	0.78±0.84 (43/52)	0.93±0.77 (44/51)	0.98±0.82 (40/55)
	Met/Met(67)	0.93±0.78 (40/27)	0.55±0.56 (40/27)	0.79±0.77 (29/38)	0.78±0.72 (33/34)	0.80±0.68 (24/24)
	P	P = 0.208 P = 0.187	P = 0.156 **P = 0.029** +	P = 0.364 P = 0.232	P = 0.423 P = 0.933	P = 0.358 P = 0.321
rs2030324	C/C (51)	0.84±0.71 (34/17)	0.43±0.51 (36/15)	0.67±0.66 (28/23)	0.64±0.66 (31/20)	0.73±0.70 (32/19)
	C/T(102)	1.16±0.90 (48/54)	0.71±0.61 (43/59)	0.82±0.85 (44/58)	0.94±0.77 (45/57)	0.96±0.78 (44/58)
	T/T (57)	1.18±0.84 (24/33)	0.79±0.76 (26/31)	0.67±0.75 (28/29)	0.94±0.73 (24/33)	0.97±0.74 (24/33)
	P	P = 0.064 **P = 0.024** +	**P = 0.009** * **P = 0.003** +	P = 0.390 P = 0.376	**P = 0.039** * P = 0.093	P = 0.166 **P = 0.045** +
rs1491850	C/C (47)	1.19±0.77 (20/27)	0.80±0.78 (22/25)	0.72±0.80 (22/25)	0.95±0.75 (19/28)	0.99±0.73 (20/27)
	C/T (92)	1.13±0.96 (43/49)	0.71±0.65 (39/53)	0.80±0.84 (41/51)	0.91±0.78 (43/49)	0.96±0.77 (39/53)
	T/T (71)	0.95±0.73 (43/28)	0.52±0.52 (44/27)	0.68±0.68 (37/34)	0.75±0.67 (38/33)	0.78±0.74 (41/30)
	P	P = 0.259 P = 0.101	**P = 0.045** * **P = 0.041** +	P = 0.618 P = 0.628	P = 0271 P = 0.369	P = 0.193 P = 0.110

* p<0.05 (ANOVA).

+ p<0.05 (χ2 test).

** two groups divided by median LDAEP.

Table 1 also lists the distribution of genotypic frequencies between subjects with low and high LDAEP, which are divided by each median LDAEP in five electrodes. The distribution of rs6265 genotypic frequencies differed significantly between subjects with high and low LDAEP at Pz (χ^2^ = 7.072, *p* = 0.029), as did those of rs2030324 at Cz, Pz, and C4 (χ^2^ = 7.422 and *p* = 0.024, χ^2^ = 11.595 and *p* = 0.003, and χ^2^ = 6.194, *p* = 0.045, respectively). However, there was no significant difference in the distribution of rs2030324 genotypic frequencies between subjects with high and low LDAEP at C3 (*p* = 0.093) and Fz (*p* = 0.376). The distribution of rs1491850 genotypic frequencies differed significantly between subjects with high and low LDAEP at Pz (χ^2^ = 6.392, *p* = 0.041). The frequencies of all three *BDNF* markers (rs6265, rs2030324, and rs1491850) at Pz were higher for subjects with a high LDAEP than for those with a low LDAEP.

The mean LDEAP was significantly higher for female subjects than for male subjects only at C3 (*t* = –2.671, *p = *0.008), with there being no gender differences in the LDAEP at Cz, Pz, Fz, and C4.

### Haplotype analysis

The overall haplotype (rs6265, rs2030324, and rs1491850) frequencies did not differ significantly between the low- and high-LDAEP groups at Cz and Pz, although the frequency of one haplotype (A-C-T) did differ significantly between the low- and high-LDAEP groups at Cz (43% vs. 22%, respectively; *p* = 0.014; Table S3, S4). However, the frequencies of the rs2030324- and rs1491850-containing haplotypes differed significantly between the low- and high-LDAEP groups at Pz (*p* = 0.017; Table 2). The frequency of one haplotype (C-T) differed significantly between the low- and high-LDAEP groups at Cz (55% vs. 42%, respectively; *p* = 0.000; Table 2).

**Table 2:** Haplotype distribution in Pz (rs2030324 and rs1491850).

Haplotype	Overall p-value	Haplotype frequencies		Permutation p value
		Low LDAEP	High LDAEP	
C-T	0.017*	0.55	0.42	0.000*
T-C		0.40	0.48	0.108
T-T		0.057	0.10	0.07

* p<0.05.

## Discussion

This was a replication study, in that it had the same design as that of Juckel and colleagues [20]. However, this is the first study to show an association between *BDNF* SNPs [rs6265(Val/Met), rs2030324, and rs1491850] and the LDAEP in an Asian population. We found that the LDAEP at Pz differed significantly among the rs1491850 and rs2030324 genotype groups (table 1), and although we did not find significant differences in the LDAEP at Pz among the rs6265 genotype groups (in ANOVA), we did observe a trend indicating that the LDAEP of the Val/Met (A/G) genotype group was higher than that of the Val/Val (G/G) genotype in post-hoc analysis (least-squares difference, *p* = 0.054). These results indicate that the serotonergic activity is lower in the Val/Met genotype group than in Val/Val. In addition, there were significant differences in the distribution of genotypic frequencies between subjects with high and low LDAEP at Pz (rs6265) (table 1). In particular, rs6265 Val/Met genotype group had more subjects with relatively high LDAEP than other groups.

Previous studies found that the response to antidepressants was better in subjects with the Met variant, which suggests that heterozygous and homozygous patients exhibit different treatment responses [25], [26]. It has also been shown that the rs6265 Val/Met heterozygous is associated with a better response to citalopram in major depression or lithium in bipolar disorder [27], [28]. Furthermore, a recent meta-analysis revealed an association between the rs6265 Val/Met polymorphism and treatment response in patients with major depressive disorder, and found that the response rate was higher in Val/Met heterozygous patients than in Val/Val homozygous patients, especially in Asian populations [29]. The results of some studies on LDAEP suggest that low serotonergic activity is a positive predictor for response to antidepressant treatment or prophylactic lithium [9], [30]. The findings from our healthy subjects are consistent with these earlier findings; namely, we found that among the three genotype groups, the mean LDAEP was highest (indicating lowest serotonergic activity) in the Val/Met genotype group. Thus, our study provides theoretical support for previous findings pertaining to the treatment response to SSRIs or lithium in Val/Met heterozygous patients with mood disorder. However, the reported Met-allele frequency of the Val66Met polymorphism has varied widely among populations, from 0% to 72%, being virtually absent in all Sub-Saharan African and some American indigenous populations [31]. Thus, the observed differences in *BDNF* between populations have implications for interpreting the conflicting association literature for psychiatric disorders [31].

There have been few studies related to psychiatric disorder in patients with the rs2030324 genotype. One haplotype containing rs2030324 was found to be associated with nicotine dependence in male Caucasians [32]. The analysis of haplotypes containing rs2030324 in another study revealed that a common four-locus haplotype is protective against schizophrenia [33]. However, no association with lithium response was revealed with the polymorphism of the rs2030324 gene [28].

The C allele for rs1491850 was found to be associated with a better response and nonresistance to treatment [34]. Another study found that the C allele for rs1491850 was associated with a better response to SSRIs in patients with obsessive-compulsive disorder (*p* = 0.002) [35]. Consistent with Kocaba’s findings, our results show that carriers of the C/C genotype exhibit a significantly higher LDAEP, indicating low serotonergic activity.

The overall haplotype (rs6265, rs2030324, and rs1491850) frequencies did not differ significantly between low- and high-LDAEP groups at Cz and Pz, although the frequency of one haplotype (A-C-T) did differ significantly between the low- and high-LDAEP groups at Cz (43% vs. 22%, respectively; *p* = 0.014). However, we found differences in the frequencies of individual haplotypes (rs2030324 and rs1491850) between the low- and high-LDAEP groups at Pz (*p* = 0.017) (table 2). The numbers of subjects with low LDAEP were more than those with high LDAEP in the C-T haplotype (C genetype for rs2030424 and T genotype for rs1491850), indicating higher central serotonergic activity. Our findings support the previously held notion that the LDAEP is related to a haplotype of the brain-derived neurotrophic factor (BDNF) gene [20]. However, the interaction patterns between LDAEP and *BDNF* polymorphism were not the same. Juckel and collegues [20] suggested that subjects with the *BDNF* haplotype G(Val)-C-T are characterized by a high LDAEP. This discrepancy can be explained in three ways. First, the interactions might differ with ethnicity. It is well known that the Korean population has a homogeneous blood line, and our results suggest that the LDAEP–*BDNF* interaction differs between Koreans and Germans. Second, our study cohort was younger and had a smaller age range (24.09±3.23 years; range, 20–32 years) than that of Juckel et al. (43±15 years; range, 19–72 years). Although there is no known association between age and LDAEP in major depressive disorder patients [9], [10], a gender-specific effect of aging on central 5-HT function has been demonstrated [36]. Third, our results were obtained from a cortical LDAEP source, while theirs was obtained from a tangential LDAEP source. It is known that single-electrode and dipole-source LDAEPs could differ significantly [37]. Thus, LDAEP results obtained from two different sources should be compared with caution.

Our results suggest that *BDNF* polymorphisms and LDAEP patterns can be used to predict altered serotonergic activity. Val/Met heterozygous mice were found to display increased depressive-like behaviors and more prominent changes in BDNF levels relative to wild-type mice, with the depressive-like behaviors able to be rescued by acute administration of desipramine [38]. Some researchers have developed heterogeneous BDNF(+/−) knockout models to study depressive-like behaviors in adult mice, due to the early postnatal lethality in homogeneous BDNF(−/−) mice [39]. Lyons and colleagues found that the BDNF(+/−) mice display behavioral abnormalities that are correlated with 5-HT dysfunction [40]. In addition, Monteggia and colleagues demonstrated that the loss of forebrain BDNF attenuates the actions of desipramine in the forced swim test, suggesting that BDNF affects the efficacy of antidepressants [41]. However, there is contrary evidence that a chronic reduction of BDNF protein content in adult BDNF(+/−) mice is not sufficient to induce neurochemical or behavioral alterations, although some investigators believe that the baseline behaviors of these mice may be difficult to interpret since they display a normal behavioral phenotype even when BDNF is expressed at half its normal level [13], [42]. Djalali and colleagues also found that the presence of BDNF is not a requirement for the survival and maturation of serotonergic neurons in vivo [43]. Thus, the relationships among BDNF, 5-HT, and depression remain controversial.

Our results showed that there were no gender differences in the LDAEP at most of electrodes (Cz, Pz, Fz, and C4) except C3. However, there was evidence that the LDAEP is modulated by gender potentially. Oliva and collegues reported that LDAEP for female healthy participants was stronger than that for male healthy participants [44]. There was contrary evidence that female depressed patients showed weaker LDAEP strength than male depressed patients [45]. In addition, there were some findings revealing no gender difference in LDAEP like our results [9], [46]. Thus, there was a still controversy about the gender difference in LDAEP.

There are several limitations to the generalization of the results of this study. First, we investigated only three SNPs. However, these three SNPs of the BDNF gene exhibit moderate-to-strong linkage disequilibrium. Second, the sample was relatively small. However, healthy subjects were carefully enrolled from a Korean population. In conclusion, the findings of our study support the view that *BDNF* polymorphisms may be involved in the LDAEP in the Korean population, although further confirmative studies are warranted.

In conclusion, it can be assumed from the results of this study that *BDNF* polymorphisms influence the LDAEP, and hence serotonin levels or serotonin activity. In particular, our study showed that subjects with the Val/Met genotype for rs6265, T/T genotype for rs2030324 or the C/C genotype for rs1491850 had a higher LDAEP, indicating lower central serotonergic activity. In addition, the numbers of subjects with low LDAEP were more than those with high LDAEP in the C-T haplotype (C genetype for rs2030424 and T genotype for rs1491850), indicating higher central serotonergic activity. It is possible that mood-disorder patients with these genotypes will exhibit a different response to medication with SSRIs or lithium. These genetic polymorphisms also seemed to be associated with the interaction between BDNF and the serotonin system.

## Supporting Information

Table S1 **Statistical analyses on demographics as well as allele frequencies of the three SNPs in BDNF gene.**
(DOC)

Table S2 **Statistical analyses on the intensity of LDAEP with genotypes of the three SNPs in BDNF gene at five electrodes (MANOVA).** In MANOVA, there was no significant difference among 3 genotype groups at 5 electrodes. There was a statistically significant difference between 2 genotype groups in rs1491850 (C/C vs T/T at Pz), rs 2030324 (C/C vs T/T, C/C vs C/T at Cz; C/C vs T/T, C/C vs C/T at Pz; C/C vs T/T, C/C vs C/T at C3, respectively, *p* = 0.037, *p* = 0.029, *p* = 0.040, *p* = 0.014,*p* = 0.033, and *p* = 0.017) (post-hoc analysis; LSD) (p<0.05).
(DOC)

Table S3 **Haplotype distribution at Cz (rs6265, rs2030324, and rs1491850).**
(DOC)

Table S4 **Haplotype distribution at Pz (rs6265, rs2030324, and rs1491850).** *p<0.05.
(DOC)
