# Supplementary material for: Association between BDNF Gene Polymorphisms and Serotonergic Activity Using Loudness Dependence of Auditory Evoked Potentials in Healthy Subjects
Source: PLoS One. 2013 Apr 9;8(4):e60340. doi: 10.1371/journal.pone.0060340 (PMC3621878; doi:10.1371/journal.pone.0060340)
Supplement: Table S1 — Statistical analyses on demographics as well as allele frequencies of the three SNPs in BDNF gene. (DOC) [file pone.0060340.s001.doc]

| **Table S1.Statistical analyses on demographics as well as allele frequencies of the three SNPs in BDNF gene.** | | | | |
| --- | --- | --- | --- | --- |
| BDNF marker | Genotype | Allele frequencies | Gender (M/F) | Age (years) |
| rs6265 | Val/Val | A 0.43/ 0.46 | 29/19 | 24.04±3.31 |
|  | Val/Met | G 0.57/ 0.54 | 44/52 | 24.15±3.15 |
|  | Met/Met |  | 38/29 | 24.03±3.34 |
|  | P | P=0.588 | P=0.183 | P=0.970 |
| rs2030324 | C/C | C 0.48/ 0.535 | 37/14 | 24.53±3.64 |
|  | C/T | T 0.52/ 0.465 | 45/58 | 24.08±3.23 |
|  | T/T |  | 29/28 | 23.70±2.83 |
|  | P | P=0.857 | P=0.003* | P=0.415 |
| rs1491850 | C/C | C 0.93/ 0.965 | 22/25 | 23.81±2.90 |
|  | C/T | T 0.07/ 0.035 | 45/48 | 24.00±3.23 |
|  | T/T |  | 44/27 | 24.38±3.45 |
|  | P | P=0.121 | P=0.150 | P=0.608 |

*p <0.05
