# Supplementary material for: Association between BDNF Gene Polymorphisms and Serotonergic Activity Using Loudness Dependence of Auditory Evoked Potentials in Healthy Subjects
Source: PLoS One. 2013 Apr 9;8(4):e60340. doi: 10.1371/journal.pone.0060340 (PMC3621878; doi:10.1371/journal.pone.0060340)
Supplement: Table S2 — Statistical analyses on the intensity of LDAEP with genotypes of the three SNPs in BDNF gene at five electrodes (MANOVA). In MANOVA, there was no significant difference among 3 genotype groups at 5 electrodes. There was a statistically significant difference between 2 genotype groups in rs1491850 (C/C vs T/T at Pz), rs 2030324 (C/C vs T/T, C/C vs C/T at Cz; C/C vs T/T, C/C vs C/T at Pz; C/C vs T/T, C/C vs C/T at C3, respectively, p = 0.037, p = 0.029, p = 0.040, p = 0.014,p = 0.033, and p = 0.017) (post-hoc analysis; LSD) (p<0.05). (DOC) [file pone.0060340.s002.doc]

| **Table S2. Statistical analyses on the intensity of LDAEP with genotypes of the three SNPs in BDNF gene at five electrodes (MANOVA).** | | | | | | |
| --- | --- | --- | --- | --- | --- | --- |
| BDNF marker | Genotype | LDAEP at Cz | LDAEP at Pz | LDAEP at Fz | LDAEP at C3 | LDAEP at C4 |
| rs6265 | Val/Val (48) | 1.03±0.17 | 0.62±0.13 | 0.57±0.16 | 0.78±0.15 | 0.82±0.16 |
|  | Val/Met (95) | 1.33±0.22 | 0.84±0.17 | 0.97±0.20 | 1.00±0.19 | 1.01±0.20 |
|  | Met/Met(67) | 0.93±0.20 | 0.64±0.16 | 0.95±0.19 | 1.03±0.18 | 0.94±0.18 |
|  | P | P=0.356 | P=0.294 | P=0.132 | P=0.337 | P=0.321 |
| rs2030324 | C/C (51) | 0.95±0.30 | 0.63±0.23 | 0.94±0.28 | 0.62±0.26 | 0.80±0.27 |
|  | C/T(102) | 1.23±0.16 | 0.67±0.13 | 1.02±0.15 | 1.02±0.14 | 0.96±0.15 |
|  | T/T (57) | 1.10±0.19 | 0.80±0.15 | 0.64±0.18 | 1.06±0.17 | 0.98±0.17 |
|  | P | P=0.710* | P=0.867* | P=0.185 | P=0.451* | P=0.983 |
| rs1491850 | C/C (47) | 1.57±0.22 | 0.99±0.17 | 0.90±0.20 | 1.27±0.19 | 1.34±0.20 |
|  | C/T (92) | 0.94±0.20 | 0.68±0.15 | 0.81±0.18 | 0.91±0.18 | 0.90±0.18 |
|  | T/T (71) | 1.04±0.19 | 0.59±0.15 | 0.87±0.18 | 0.80±0.17 | 0.74±0.17 |
|  | P | P=0.098 | P=0.516* | P=0.316 | P=0.671 | P=0.418 |

In MANOVA, there was no significant difference among 3 genotype groups at 5 electrodes. There was a statistically significant difference between 2 genotype groups in rs1491850 (C/C vs T/T at Pz), rs 2030324 (C/C vs T/T, C/C vs C/T at Cz; C/C vs T/T, C/C vs C/T at Pz; C/C vs T/T, C/C vs C/T at C3, respectively, *p*=0.037, *p*=0.029, *p*=0.040, *p*=0.014,*p*=0.033, and *p*=0.017) (post-hoc analysis; LSD) (p<0.05).
