# Supplementary material for: Association between BDNF Gene Polymorphisms and Serotonergic Activity Using Loudness Dependence of Auditory Evoked Potentials in Healthy Subjects
Source: PLoS One. 2013 Apr 9;8(4):e60340. doi: 10.1371/journal.pone.0060340 (PMC3621878; doi:10.1371/journal.pone.0060340)
Supplement: Table S3 — Haplotype distribution at Cz (rs6265, rs2030324, and rs1491850). (DOC) [file pone.0060340.s003.doc]

| **Table S3. Haplotype distribution at Cz (rs6265, rs2030324, and rs1491850).** | | | | |
| --- | --- | --- | --- | --- |
| Haplotype | Overall p-value | Haplotype frequecies | | Permutation p value |
|  |  | Low LDAEP | High LDAEP |  |
| A-C-T | 0.124 | 0.43 | 0.22 | 0.014* |
| G-T-C |  | 0.27 | 0.23 | 0.789 |
| A-T-C |  | 0.12 | 0.25 | 0.123 |
| G-C-T |  | 0.11 | 0.20 | 0.306 |
| G-T-T |  | 0.035 | 0.061 | 0.334 |
| A-T-T |  | 0.032 | 0.033 | 0.978 |

*p <0.05
