# Supplementary material for: Association between BDNF Gene Polymorphisms and Serotonergic Activity Using Loudness Dependence of Auditory Evoked Potentials in Healthy Subjects
Source: PLoS One. 2013 Apr 9;8(4):e60340. doi: 10.1371/journal.pone.0060340 (PMC3621878; doi:10.1371/journal.pone.0060340)
Supplement: Table S4 — Haplotype distribution at Pz (rs6265, rs2030324, and rs1491850). *p<0.05. (DOC) [file pone.0060340.s004.doc]

| **Table S4. Haplotype distribution at Pz (rs6265, rs2030324, and rs1491850).** | | | | |
| --- | --- | --- | --- | --- |
| Haplotype | Overall p-value | Haplotype frequencies | | Permutation p value |
|  |  | Low LDAEP | High LDAEP |  |
| A-C-T | 0.485 | 0.40 | 0.30 | 0.209 |
| G-T-C |  | 0.26 | 0.29 | 0.652 |
| A-T-C |  | 0.13 | 0.19 | 0.442 |
| G-C-T |  | 0.15 | 0.12 | 0.617 |
| G-T-T |  | 0.030 | 0.070 | 0.097 |
| A-T-T |  | 0.028 | 0.033 | 0.787 |
